# *Incomer*, a DD36E family of *Tc1/mariner* transposons newly discovered in animals

**DOI:** 10.1186/s13100-019-0188-x

**Published:** 2019-11-23

**Authors:** Yatong Sang, Bo Gao, Mohamed Diaby, Wencheng Zong, Cai Chen, Dan Shen, Saisai Wang, Yali Wang, Zoltán Ivics, Chengyi Song

**Affiliations:** 1 Institute of Animal Mobilome and Genome, College of Animal Science & Technology, Yangzhou University, Yangzhou, 225009 Jiangsu China; 2 Division of Medical Biotechnology, Paul Ehrlich Institute, 63225 Langen, Germany

**Keywords:** *Tc1/mariner* transposons, DD36E, Horizontal transfer

## Abstract

**Background:**

The *Tc1/mariner* superfamily might represent the most diverse and widely distributed group of DNA transposons. Several families have been identified; however, exploring the diversity of this superfamily and updating its classification is still ongoing in the life sciences.

**Results:**

Here we identified a new family of *Tc1/mariner* transposons, named *Incomer* (*IC*), which is close to, but distinct from the known family DD34E*/Tc1*. *ICs* have a total length of about 1.2 kb, and harbor a single open reading frame encoding a ~ 346 amino acid transposase with a DD36E motif and flanked by short terminal inverted repeats (TIRs) (22–32 base pairs, bp). This family is absent from prokaryotes, and is mainly distributed among vertebrates (141 species of four classes), including Agnatha (one species of jawless fish), Actinopterygii (132 species of ray-finned fish), Amphibia (four species of frogs), and Mammalia (four species of bats), but have a restricted distribution in invertebrates (four species in Insecta and nine in Arachnida). All *ICs* in bats (*Myotis lucifugus*, *Eptesicus fuscus*, *Myotis davidii*, and *Myotis brandtii*) are present as truncated copies in these genomes, and most of them are flanked by relatively long TIRs (51–126 bp). High copy numbers of miniature inverted-repeat transposable elements (MITEs) derived from *ICs* were also identified in bat genomes. Phylogenetic analysis revealed that *ICs* are more closely related to DD34E*/Tc1* than to other families of *Tc1/mariner* (e.g., DD34D*/mariner* and DD × D*/pogo*), and can be classified into four distinct clusters. The host and *IC* phylogenies and pairwise distance comparisons between *RAG1* genes and all consensus sequences of *ICs* support the idea that multiple episodes of horizontal transfer (HT) of *ICs* have occurred in vertebrates. In addition, the discovery of intact transposases, perfect TIRs and target site duplications of *ICs* suggests that this family may still be active in Insecta, Arachnida, frogs, and fish.

**Conclusions:**

Exploring the diversity of *Tc1/mariner* transposons and revealing their evolutionary profiles will help provide a better understanding of the evolution of DNA transposons and their impact on genomic evolution. Here, a newly discovered family (DD36E/*Incomer*) of *Tc1/mariner* transposons is described in animals. It displays a similar structural organization and close relationship with the known DD34E/*Tc1* elements, but has a relatively narrow distribution, indicating that DD36E/*IC* might have originated from the DD34E/*Tc1* family. Our data also support the hypothesis of horizontal transfer of *IC* in vertebrates, even invading one lineage of mammals (bats). This study expands our understanding of the diversity of *Tc1/mariner* transposons and updates the classification of this superfamily.

## Background

Fragments of DNA sequences, which can autonomously replicate and translocate between chromosomes, are called transposable elements (TEs) or transposons. The first transposon was discovered by Barbara McClintock in maize [1]. They were subsequently detected in various organisms, such as bacteria, fungi, and insects. Based on their mechanism of transposition, TEs can be divided into two major classes: class I transposons transpose by RNA (also called retrotransposons); and class II transposons transpose by DNA (also called DNA transposons). Class II transposons can be further divided into three subcategories: the classical “cut-and-paste” DNA transposons, “rolling circle” DNA transposons, and “self-synthesizing” DNA transposons [2]. For a long time, transposons were designated as “junk DNA” in the genome and ignored. However, with the completion of large-scale genome sequencing projects, transposons have been found to exist in almost all genomes. It is now believed that TEs play important roles in genomic evolution and are regarded as important factors in determining genome expansion. They can simultaneously modify gene structures, provide sources of regulatory sequences [3, 4], and have important impacts on the structure and evolution of the genes of eukaryotes [5, 6].

*Tc1/mariner* is an important superfamily of “cut-and-paste” transposons, which was first discovered in *Drosophila mauritiana* [7, 8]. Elements in this superfamily are generally 1300–2400 base pairs (bp) in size and encode a 340-amino acid (aa) transposase that is flanked by TIRs and a TA target site duplication (TSD) at each end [9]. *Tc1/mariner* transposases contain a DNA-binding domain (DBD), which harbors two helix–turn–helix (HTH) motifs [10], a conserved GRPR-like sequence between the two HTH motifs [11], and a conserved catalytic amino acid triad motif (DDE/D), which usually interacts with a divalent cation (Mg^+ 2^ or Mn^+ 2^) to perform the biochemical steps of the transposition reaction [12]. The distance between the first two “D” amino acids is variable across different transposase families, while the distance between the “D” and the third “D/E” is highly conserved. Accordingly, the length of this spacer has been used to characterize this transposase family [13]. Regarding variations of the DDE/D signature motif, *Tc1/mariner* elements have been classified into eight distinct families: DD34E/*Tc1*, DD34D/*mariner*, DD37E/*TRT*, DD37D/*maT*, DD39D, DD × D/*pogo*, DD41D, and DD × E [14]. Although many *Tc1/mariner* transposons have been identified in nature, only a few naturally active *Tc1/mariner* transposons have been discovered, such as *Thm3* [15], *Tc1* [16], *Tc3* [17], and *Mos1* [18]. This is because insertion of the transposon results in instability of the genome; therefore, long-term purifying natural selection, genetic drift, and mutations can result in gradual inactivation or even disappearance of transposons within the host genome [19, 20]. In addition, studies have shown that horizontal transfer (HT) of transposons is an important way to avoid inactivation and extinction. The HT of transposons between species is considered to be an important driver of genomic variation and biological innovation [21]. Almost all kinds of eukaryotic superfamily TEs have been proved to be capable of HT [21], while the *Tc1/mariner* superfamily seems to be more prone to this behavior [22]. More than 1200 HT events of *Tc1/mariner* have been reported to date [23].

Gaining further insight into the evolutionary profile of *Tc1/mariner* transposons will provide a better understanding of DNA transposon evolution and their impact on genome evolution. Here, we uncovered a new family of *Tc1/mariner* transposons, which is closely related to the DD34E*/Tc1* family, but forms a distinct clade and harbors a DD36E motif in its DDE domain. We also report the taxonomic distribution of *ICs*, describe the structural organization of these elements, and provide evidence to support the occurrence of HT among vertebrates, and the invasion of this family in one lineage of mammals (bats).

## Results

### Taxonomic distribution of *ICs*

**Fig. 1:**
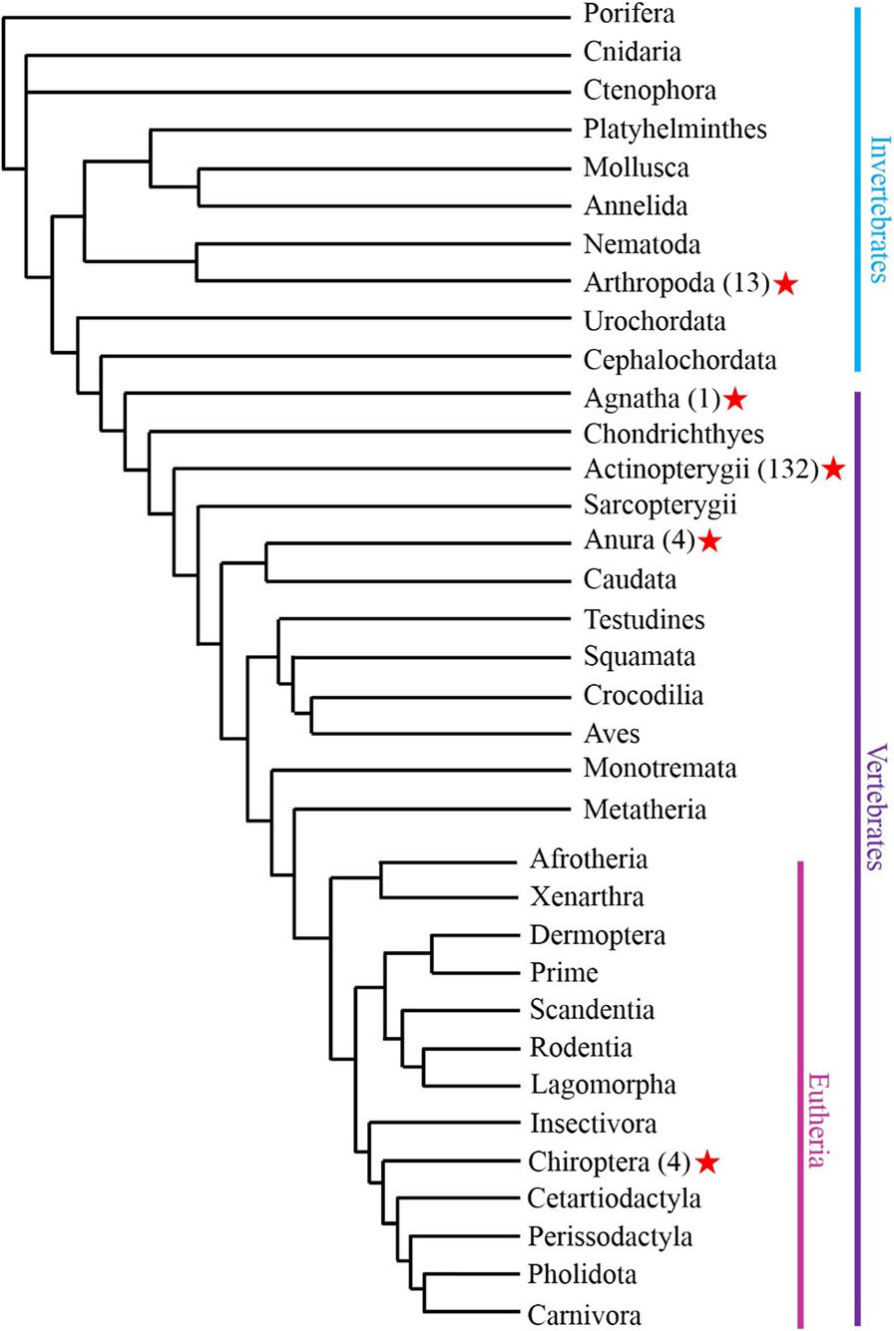
Taxonomic distribution of *IC* elements among Eukaryota. *IC* elements identified in the branches are shown as red stars, and the numbers in parentheses represent the number of species that with *IC* elements in each branch

Using TBLASTN searching (https://blast.ncbi.nlm.nih.gov/Blast) with the DD34E references (*Passport*, *Prince*, *Quetzal*, and *Sleeping Beauty*) as queries, we identified an intact *Tc1/mariner*-like transposon in *Rhinella marina*, where it harbors a newly identified transposase family with a DD36E motif, which is close to, but distinct from the previously known family of DD34E*/Tc1*. We named this newly discovered member of the *Tc1/mariner* superfamily *Incomer* or DD36E*/IC*. To investigate the evolutionary profile of this family, a TBLASTN search against all the available organism genomes of prokaryotes (bacteria and archaea) and the eukaryotes (Protozoa, Animalia, Fungi, Plantae, and Chromista) deposited at the National Center for Biotechnology Information (NCBI) database (https://www.ncbi.nlm.nih.gov) was performed using the *IC* transposase (346 aa) of *R. marina* as the query term. The obtained *IC* transposases were in turn used as query terms to identify more *IC* elements. These searches revealed that this family was absent in prokaryotes, and displayed a narrow distribution in eukaryotes with a main distribution in the animal kingdom (Fig. 1 and Additional file 1: Table S1). Further analysis revealed that the *IC* transposons were present in invertebrate and vertebrate groups, but that *IC* showed a very narrow distribution in invertebrates, only present in two classes (four species of Insecta and nine species of Arachnida) among the Arthropoda. In vertebrates, *IC* transposons were found in 141 species among four classes, including Agnatha (one species of jawless fish), Actinopterygii (132 species of ray-finned fish), Amphibia (four species of frogs), and Mammalia (four species of bats). This family did not undergo significant expansion in most classes of animals, but radiated in the Actinopterygii, where it displays an extensive distribution in 132 species of 38 orders compared with other classes (Additional file 2: Figure S1). In addition, most of the *IC* transposons are present as truncated copies: thus, in Actinopterygii, more than half of the species (76/132) contain full-length *IC* elements (with two detectable TIRs), but only 33 species contain intact *IC* copies (with an intact transposase and two detectable TIRs). Among the Anura, four species contain full-length *ICs* and three of them harbor intact copies of *ICs*. Intact *IC* copies were also detected in one species of Agnatha, while all *ICs* in the four species (*Myotis lucifugus*, *Eptesicus fuscus*, *M. davidii*, *M. brandtii*) of the Chiroptera are present as truncated copies and an intact copy was not detectable. Among the Arthropoda, four species of Insecta and seven species of Arachnida contain full-length *IC* copies, with four and three species harboring intact *IC* elements, respectively (Table 1). The discovery of intact transposases, perfect TIRs, and TSDs of *ICs* suggests that this family could still be active in insects, Arachnida, frogs, and fish.

**Table 1:** Taxonomic distribution of *IC* elements

Taxonomic distribution	Mammalia (Bat)	Amphibia (Frog)	Actinopterygii	Myxini	Arachnida	Insecta
Number of species containing IC	4	4	132	1	9	4
Number of species containing full IC	4	4	76	1	7	4
Number of Species containing intact IC	0	3	33	1	3	3
Length of full IC	1005–1487	1194–1225	1099–1467	1226	1200–1234	1213–1226
Length of intact IC	N	1194–1225	1169–1229	1226	1220–1224	1217–1226
Transposase length of intact IC	N	346	314–356	346	346–382	346–366
TIR length of intact IC	N	28–29	24–32	28	25–27	25–28
TSD	TA	TA	TA	TA	TA	TA

In addition, the copy number of *ICs* in the genomes of different organisms varies dramatically, from only one copy (> 90% of identity and > 1000 bp in length) in some organisms (e.g., *Seriola dumerili*, *Amphilophus citrinellus*, *Gadiculus argenteus*, and *Xenocatantops brachycerus*) to several thousand copies in a few species including *R. marina*, *Esox lucius*, and *Clitarchus hookeri*. *ICs* have undergone significant amplification in *R. marina*; i.e., more than 5000 copies of *ICs* were detected and more than half of them are full-length copies (2949 copies). *ICs* are also enriched in the genomes of *E. lucius* (5418 copies) and *C. hookeri* (3, 956 copies) (Additional file 1: Table S1). Previous studies revealed that both the *R. marina* and *C. hookeri* genomes contain high repeat contents, with 63.9 and 51.6% of their genomes covered by repeats, respectively [24, 25]. These data indicate that some organisms might be more susceptible to HT of transposons and tend to enrich repeated copies in their genomes.

### Structural organization of *ICs*

**Fig. 2:**
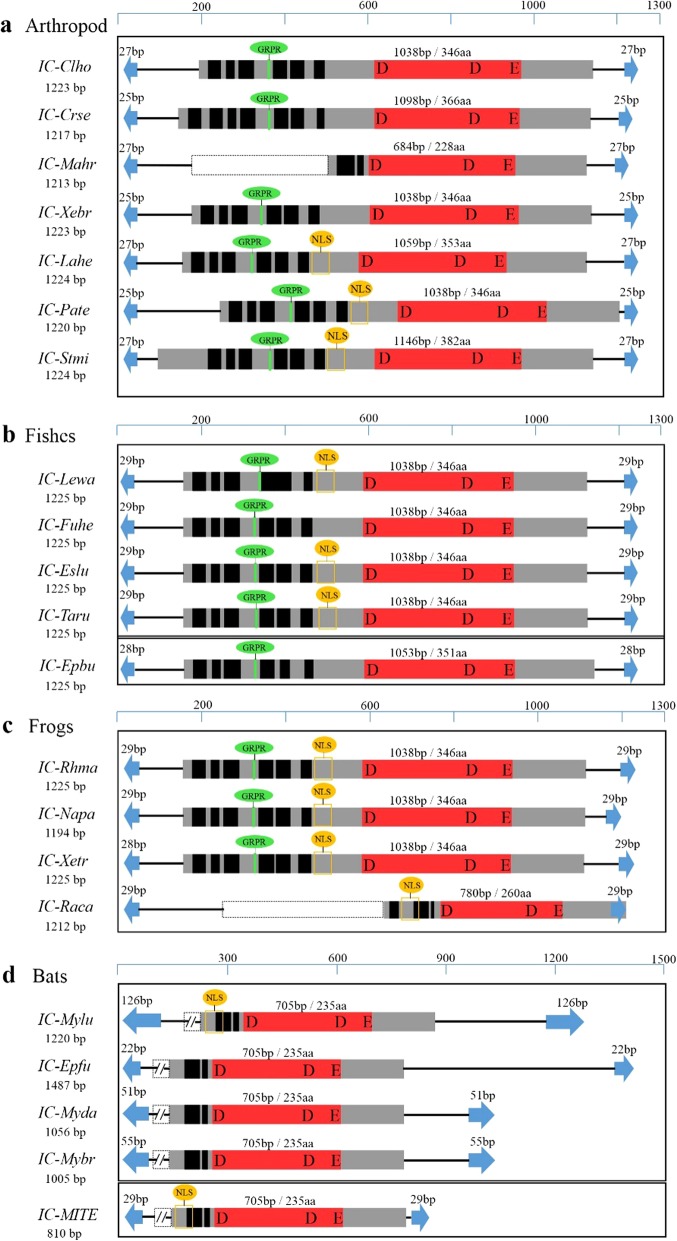
Structural organization of *ICs*. The blue arrows represent TIRs; the black rectangles represent HTH motifs; green rectangles represents GRPR sequences; the orange wire frame represents the NLS; the red rectangles represents catalyt*ic* domains, D:aspartic acid, E:glutamic acid; and the gray region represents transposases. The dotted box represents the portion of the transposases that can be deleted in the particular species. *Clho:Clitarchus hookeri; Crse:Cryptotermes secundus;Mahr: Machilis hrabei; Xebr:Xenocatantops brachycerus; Lahe:Latrodectus hesperus; Pate:Parasteatoda tepidariorum; Stmi:Stegodyphus mimosarum; Lewa:Leuciscus waleckii; Fuhe:Fundulus heteroclitus; Eslu:Esox lucius; Taru:Takifugu rubripes; Epbu:Eptatretus burgeri; Rhma:Rhinella marina; Napa:Nanorana parkeri; Raca:Rana catesbeiana; Xetr:Xenopus tropicalis. Mylu: Myotis lucifugus;Epfu: Eptesicus fuscus; Myda:Myotis davidii; Mybr:Myotis brandtii*

The structural organization of *ICs* was found to be highly conserved across different classes of animals including insects, Arachnida, fish, frogs, and bats. Most intact *IC* transposons had a total length of about 1.2 kb and harbored a single open reading frame (ORF) encoding a protein of about 346 aa in length (range 335–382 aa) flanked by short TIRs (22–32 bp) (Table 1 and Fig. 2). The *IC* elements were found to be flanked by TA target site duplication (Table 1). The intact *IC* transposon in *R. marina*, representing a typical structure of this family, is 1225 bp long, encoding a 346 aa transposase and flanked by 29 bp TIRs (Fig. 2). Several conserved motifs, including six predicted helices in two HTHs and GRPR in the N-terminal DBD, and a nuclear localization sequence (NLS), which are characteristic of *Tc1/mariner* transposases [11], were identified in most *IC* transposases by in silico prediction. The DDE signature and its spacing (36 aa) in the DDE domain seems to be highly conserved across the *IC* family (Fig. 2 and Additional file 2: Figure S2). All *ICs* in the four genomes of bats (*M. lucifugus*, *E. fuscus*, *M. davidii*, and *M. brandtii*) presented as truncated copies; the longest *ICs* in these species are 1220, 1487, 1056, and 1005 bp, respectively, and encode a truncated transposase (235 aa) containing a partial DBD and a DDE domain. Moreover, the TIR lengths of bat *ICs* also vary slightly in three bat species compared with the *IC* TIRs in the genomes of other organisms, with 126 bp in *M. lucifugus*, 51 bp in *M. davidii*, and 55 bp in *M. brandtii* (Fig. 2). We also found a high copy number of miniature inverted-repeat transposable elements (MITEs) derived from *ICs* in the four bat genomes and most of these have a length of about 810 bp. Some MITE copies also encode the truncated transposase (235 aa) (Fig. 2 and Table 2).

**Table 2:** Incomer in bats

Species	TE name	Length	Transposase length	Copy number	% Ave. Divergence ±SE
*M. lucifugus*	IC-MITE1-Mylu	1220	235	407	4.4 ± 0.08
	IC-MITE2-Mylu	810	235	951	5.2 ± 0.09
*E. fuscus*	IC-MITE1-Epfu	1487	235	1	NA^a^
	IC-MITE2-Epfu	810	235	367	3.9 ± 0.05
*M. davidii*	IC-MITE1-Myda	1056	235	4	NA
	IC-MITE2-Myda	810	235	147	5.3 ± 0.6
*M. brandtii*	IC-MITE1-Mybr	1005	235	48	4.0 ± 0.15
	IC-MITE2-Mybr	810	235	215	3.5 ± 0.06

*SE* standard error

^a^ Average percent divergence could not be determined for full-length *IC* elements due to low copy number

### Phylogenetic analysis and evidence for multiple HT events of *ICs*

**Fig. 3:**
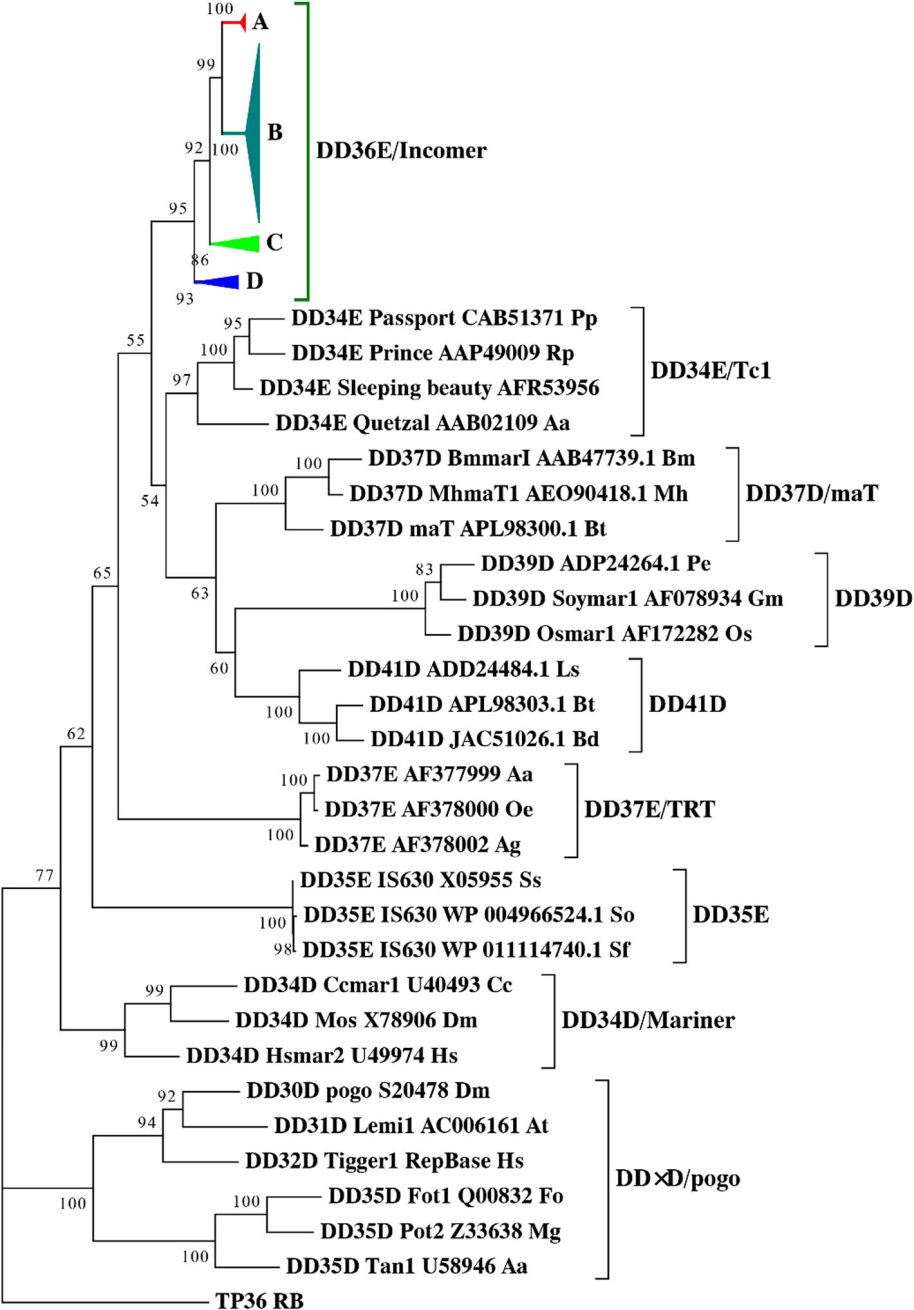
Phylogenetic tree of *IC* elements identified in this study with eight other members of the *Tc1/mariner* superfamily based on their transposases. Bootstrapped (1000 replicates) phylogenetic trees were inferred by using the Maximum Likelihood method in IQ-TREE [27]. Each sequence (except the DD37E and DD41D subclasses) contains the name of the transposon, the gene sequence number corresponding to the transposon, and the Latin abbreviation of the species in which the transposon is located. *Pp:Pleuronectes platessa;Rp:Rana pipiens; Aa:Anopheles albimanus; Bm:Bombyx mori; Mh:Misgolas hubbardi; Bt:Bactrocera tryoni; Pe:Phyllostachys edulis; Os:Oryza sativa; Gm:Glycine max; Ls:Lepeophtheirus salmonis; Bd:Bactrocera dorsalis; Aa:Aedes atropalpus; Oe:Ochlerotatus epactius; Ag:Anopheles gambiae; Serratia odorifera Sf:Shigella flexneri; Ss:Shigella sonnei; Hs:Homo sapiens; Cc:Ceratitis capitata; Dm:Drosophila mauritiana; Dm:Drosophila melanogaster; At:Arabidopsis thaliana; Aa:Aspergillus awamori; Fo:Fusarium oxysporum; Mg:Magnaporthe grisea*

**Fig. 4:**
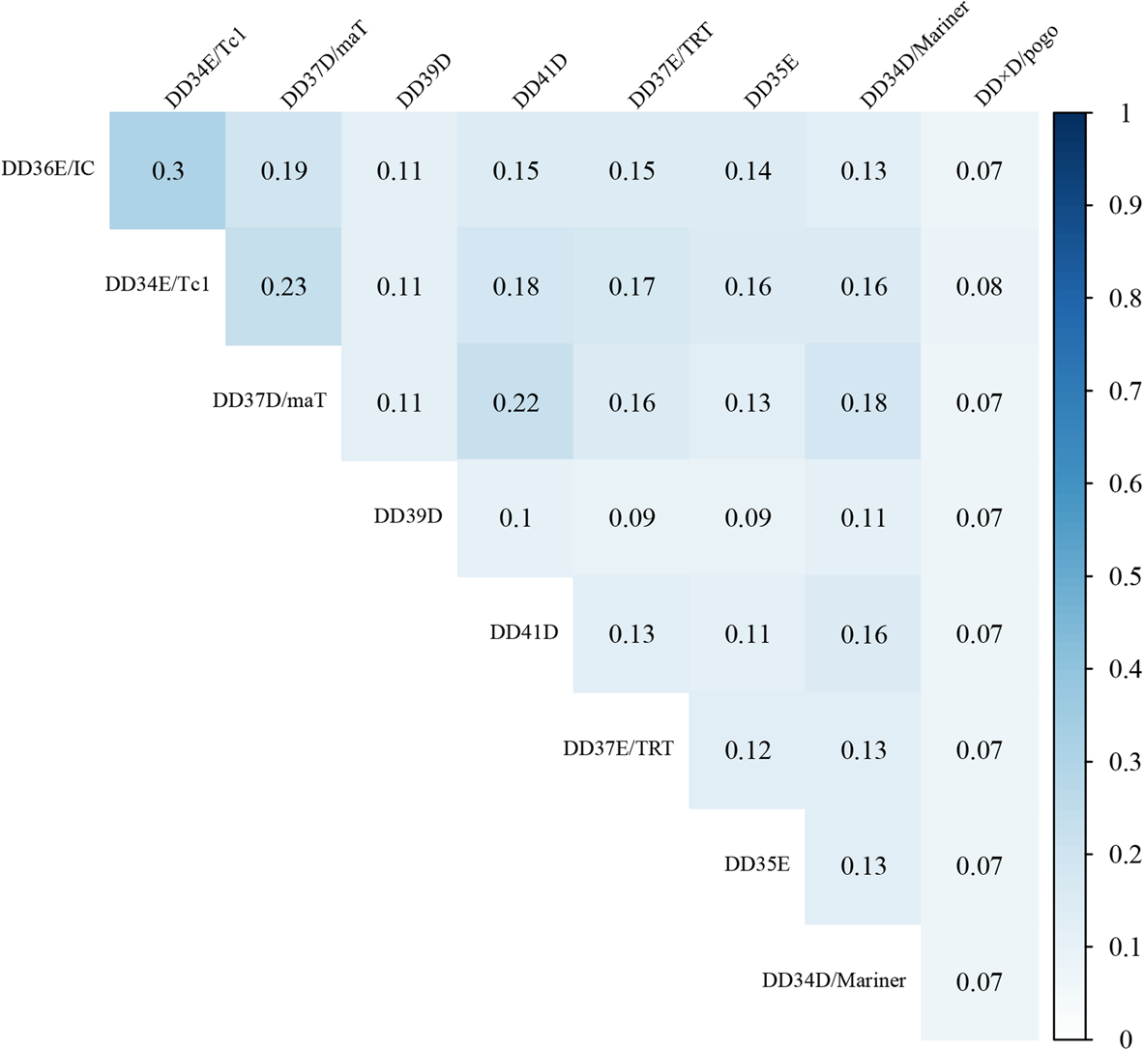
Sequence identities between *IC* family and eight other families. The sequence identities were measured by pairwise comparisons of full-length transposases

To accurately establish the evolutionary relationships of the *IC* elements that we identified, the conserved DDE domain of the identified *IC* transposases were aligned to the 28 known DNA transposases representing the eight families in the *Tc1/mariner* superfamily based on MAFFT v 7.310 [26]. The alignment was used for phylogenetic analysis using the maximum-likelihood method implemented in IQ-TREE [27], and the TP36_RB, which is an insertion element family identified in bacteria, and close to the *Tc1/mariner* transposases [28] was used as the outgroup. The polygenetic tree confirmed that all these elements identified belong to the DD36E*/IC* family, which is more closely related to the DD34E*/Tc1* family than to other families of *Tc1/mariner* (e.g., DD34D*/mariner* and DD × D*/pogo*; Fig. 3). The deduction is also well supported by the highest transposase sequence identity between these two families (Fig. 4), and further confirmed by the phylogenetic tree generated by using the alignment of the full-length transposases (Additional file 2: Figure S3).

**Fig. 5:**
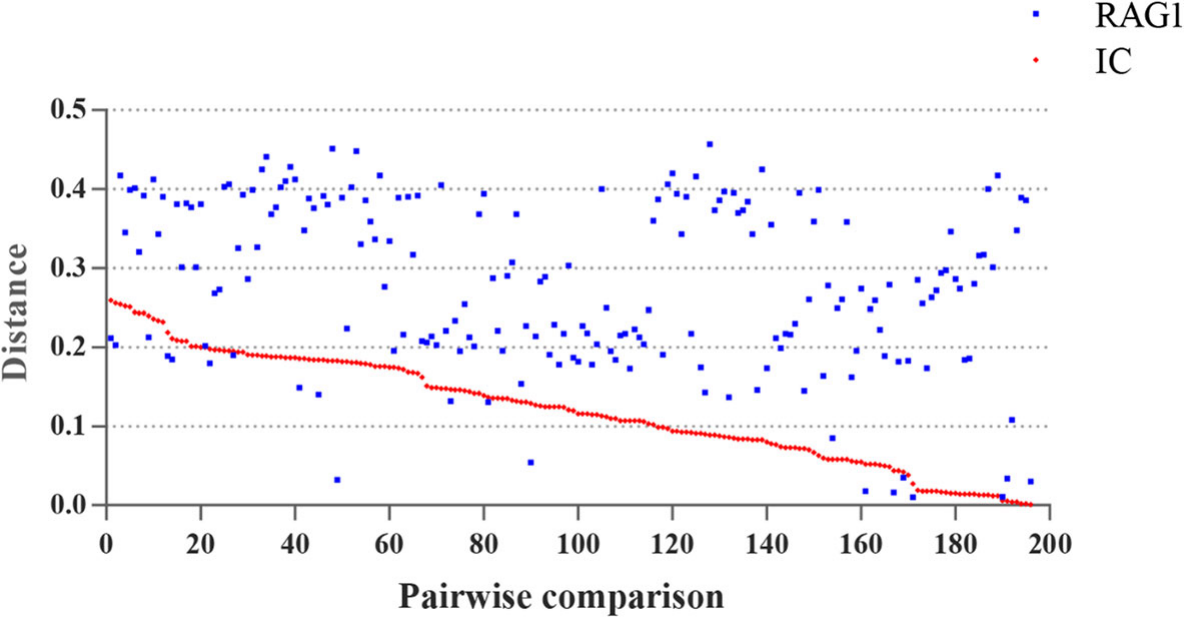
Pairwise distances of *IC* elements and *RAG1*. The distances are obtained from all possible pairwise comparisons (*n* = 196; labeled on the x axis) between the four (Cluster A) and 20 (Cluster B) species in which *IC*s were identified and complete. The coding sequence (CDS) regions of the *RAG1* gene in the NCBI database are available (Additional file 7: Text S3 and Additional file 8: Text S4)

The above phylogeny showed that *IC* elements identified in this study could be classified into four major clusters: Cluster A includes five species (one frog and four bats); Cluster B includes 59 species (three frogs and 56 fishes); Cluster C includes five species (two fishes and three insects); and Cluster D includes four species (all insects; Additional file 2: Figure S4). Phylogenetic analysis also suggested that the host and *IC* phylogenies were incongruent (Fig. 3 and Additional file 2: Figure S5), which implied that *IC* elements might have been exposed to several episodes of HT. To test this, pairwise distances between recombination-activating gene 1 (*RAG1*) and all consensus sequences of *ICs* in vertebrate were calculated and compared, which are usually used to infer the HT events of transposons in vertebrates [29, 30]. As illustrated in Fig. 5, for almost all (177/196) pairwise comparisons, the distances computed for *IC* (average 0.121; standard deviation, SD ± 0.067; range 0.001–0.259) are much lower than those calculated for *RAG1* (average 0.278; SD ± 0.106; range 0.009–0.457) (Additional files 3: Table S2). TEs are known to evolve neutrally after insertion in a host genome [31]; thus, TE distances between taxa are expected to be higher than distances between orthologous genes and to evolve faster than the host genes that evolve under purifying selection because of functional constraints under vertical transmission of the TEs. The low pairwise *IC* distances, combining deep divergence times, with most species involved sharing a last common ancestor more than 110 million years ago (Ma), indicate multiple HT events of *ICs* in vertebrates.

**Fig. 6:**
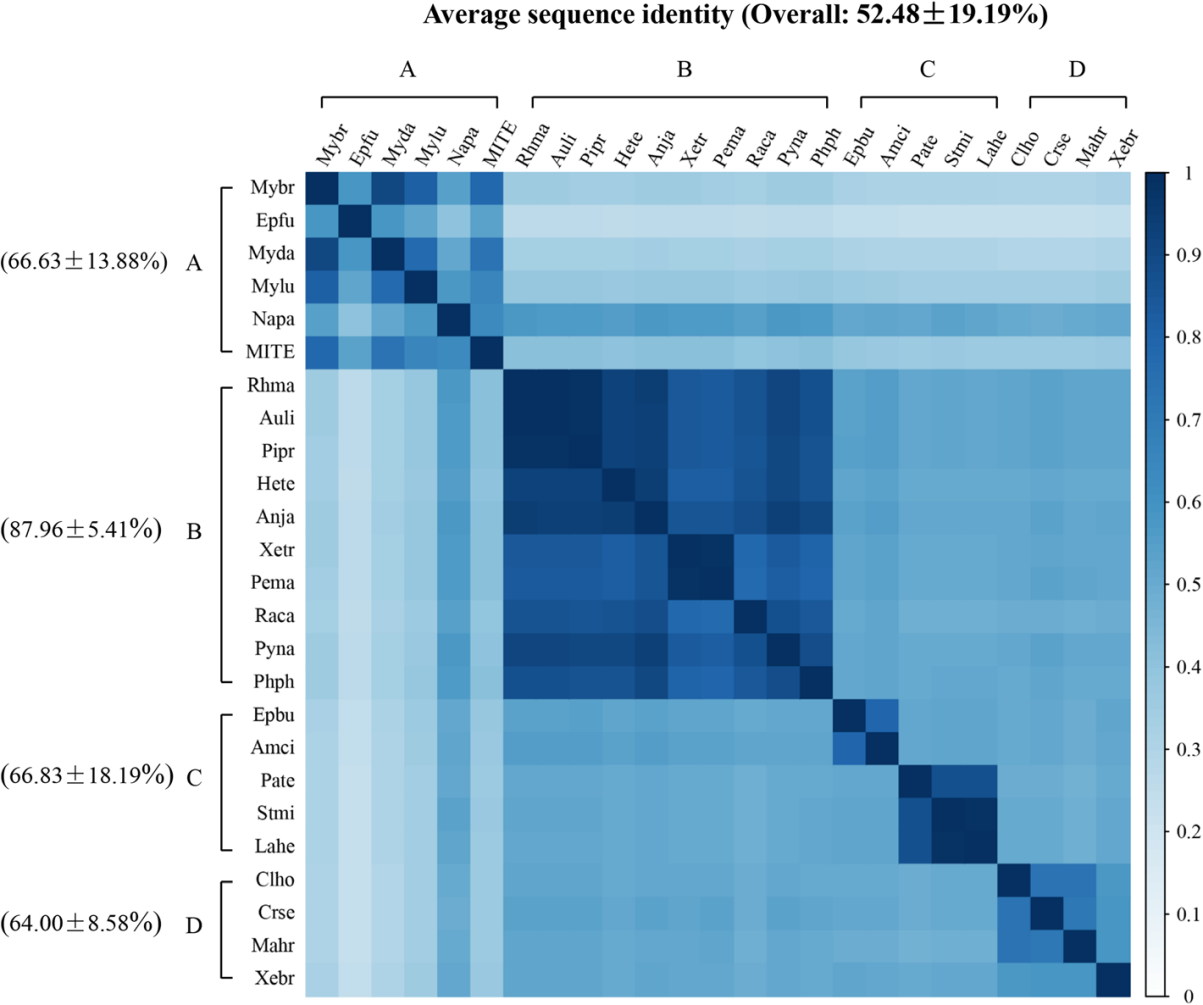
Sequence identities between *IC* elements among species. The sequence identities were measured by pairwise comparisons of full-length *IC* consensus sequences. *Auli: Austrofundulus limnaeus; Pipr: Pimephales promelas; Hete: Helostoma temminkii; Anja: Anguilla japonica; Pema: Periophthalmus magnuspinnatus; Pyna: Pygocentrus nattereri; Phph: Phycis phycis*

To further illustrate the HT profiles of *IC* elements in animal, we compared the average sequence identities of *IC* elements across species and clusters, which was summarized in Fig. 6. And the sequence identity matrix showed that most *IC*s in cluster B represent higher sequence identities (> 78%; average 87.96 ± 5.41%) between species, indicating that the cluster B may represent a very young HT event, while high and low sequence identities of *IC*s between species co-exist in cluster A, C, and D, indicating that these cluster may experience young and old invasions of *ICs*.

## Discussion

### Expanding the diversity of the *Tc1/mariner* superfamily

Compared with other DNA transposons, the *Tc1/mariner* superfamily might not only be the most widely distributed group of transposons in nature, but also displays the highest diversity. Phylogenetic analyses based on the distinct “DDE/D” signatures of transposases from diverse organisms suggest that *Tc1/mariner* transposons comprise at least eight families in eukaryotes: (1) DD34D/*mariner*; (2) DD39D; (3) DD37E; (4) DD34E/*Tc1*; (5) DD37D; (6) DD35E; (7) DD × D/*pogo*; and (8) DD41D [14, 32]. Here, we expanded the diversity of the *Tc1/mariner* superfamily and identified the ninth family (DD36E/*IC*) of these transposons, discontinuously distributed in 13 species of invertebrates and 141 species of vertebrates. The structure organizations of *ICs* are very similar to the known DD34E*/Tc1* elements and present all the hallmark features of *Tc1*-like elements, including the existence of a transposase of about 346 aa in length, a DDE motif, two HTH motifs in the DNA-binding domains, TIRs, and a TSD TA at each end. However, the spacing of the DDE motif within the catalytic domain of *IC* is unique, with 36 aa (DD36E) separating the second aspartic acid and the glutamic acid residues. The phylogenetic analyses place *IC* elements in a distinct group separate from other known families of *Tc1/mariner* transposons, which suggests that these elements constitute a newly discovered family within the *Tc1/mariner* superfamily. Furthermore, the increasing numbers of newly discovered families of the *Tc1/mariner* superfamily as more genome sequencing data becomes available [29, 33] indicates that the diversity of *Tc1/mariner* superfamily may be far greater than currently known.

### Origin of *IC*s and relationship to the DD34E /*Tc1* family

The host of the earliest branching *IC* clade was the phylum Arthropoda, the next earliest branching clade included elements from Agnatha. However, here the phylogenetic trees generated by using both of the full-length transposases and the DDE domains indicated that the *IC* family is closest to the DD34E*/Tc1* family; thus, an origin in the DD34E*/Tc1* family for the entire group of *ICs* in metazoans is more plausible, given that several DD34E*/Tc1* elements, such as *Minos* [34], *Bari* [35], *S* element [36], *Quetzal* [37], and *Topi* [38], were also identified in Arthropoda. To date, diverse DD34E/*Tc1* elements have been identified and described [34–40]; the DD34E*/Tc1* family seems to display a more extensive distribution than DD36E*/IC* and can be classified into several subfamilies, such as *Passport*-like, *Frog Prince*-like, *SB*-like, *Bari*-like, *Minos*-like [24], and *Gambol* [41], although the intra-group classification of DD34E*/Tc1* is still ambiguous. The structural organization of *ICs* is very similar to some DD34E/*Tc1* elements identified in the neoteleost genomes of Actinopterygii [32], *Bari* (*Drosophila melanogaster*) [35], and *Topi* (*Anopheles gambiae*) [38] identified in Arthropoda, where these families are relatively shorter in total length (about 1200 bp) and contain a single ORF (about 340 aa) flanked by short TIRs (about 30 bp). The structural organization of transposases, including the protein motifs of six helices, GRPR, and NLS in the DBD domain, between *ICs* and DD34E*/Tc1* is very similar as well [32, 35, 38]. Furthermore, our data also indicated that the *IC* family and DD34E/*Tc1* have the highest transposase sequence identity compared to other families. Taken together, these data indicate that the DD36E*/IC* might originate from the DD34E*/Tc1* family.

### Distribution and activity of *IC*s in animals

Compared with the DD34E*/Tc1* family, which displays extensive expansion in invertebrates and vertebrates [32, 41, 42], even among fungi [43], *ICs* show a very relatively narrow distribution in invertebrates and are only present in 13 species of two classes (Insecta and Arachnida) of Arthropoda. In vertebrates, *ICs* were detected in 141 species of four classes (Agnatha, Actinopterygii, Amphibia, and Mammalia). *ICs* display a slight burst in the Actinopterygii with an expansion into 132 species of 38 orders and have even invaded the mammalian lineage (Chiroptera) lineage, that has been suggested to be more susceptible to HT of transposons than other groups, and have experienced HT events of most DNA transposons (*hATs*, *piggyBacs*, *Tc1/mariner*, and *Helitron*) [44]. Here we provide evidence to support another HT event from a newly discovered family of *Tc1/mariner* transposons in bats, suggesting that some DNA transposons tend to show recurrent invasion of some mammalian lineages. This was confirmed in the evolution of the *hAT* superfamily, which was also found to undergo repeated HT events in mammals [30]. These data also indicate that HT of DNA transposons has contributed significantly to shaping and diversifying the genomes of multiple mammalian species, although HT of DNA transposons is relatively rare in mammals. In addition, the taxonomic distribution of *ICs* revealed by this study might have been underestimated, because of inefficient sequencing or assembly technologies, or the unavailability of some genome sequences.

Several families of *Tc1/mariner* have been suggested to be active (e.g., *Tc1*, *TRT*, *Tana1*, and *pogo*) [15, 29, 32, 39, 45] and some of them have been shown to transpose experimentally, such as *Passport* and *Thm3* in fish [15, 39]. Here, intact copies of *ICs* were identified in 33 species of bony fish, three species of frogs, one species of jawless fishes, and seven species of Arthropoda. They did not harbor internal stop codons or frameshift mutations and presented all expected functional domains, as well as intact TIRs, combining the narrow taxonomic distribution of this family, suggesting that *IC* is young and might be associated with current or recent activity in these host genomes.

## Conclusions

Our results represent the first in silico evidence for a newly identified family (DD36E/*IC*) of the *Tc1/mariner* superfamily and uncover the evolutionary landscape of this family in nature. This family is about 1200 bp in total length, encoding a transposase of ~ 346 aa flanked by short TIRs (about 30 bp), and mainly distributed in vertebrates (141 species), with a restricted presence in invertebrates (13 species). This family can be subdivided into four distinct clusters based on the catalytic domain signature DD36E. Based on structural organization, protein motifs and phylogenetic analyses, the *IC* transposons are closely related to DD34E*/Tc1* elements, indicating a recent common ancestor. Furthermore, evidence for HT events in vertebrates is well supported for this family. We have also demonstrated the presence of *IC* in the bat lineage of mammals. We propose an update of the classification of the *Tc1/mariner* superfamily and illustrate the evolutionary relationships among these distinct families.

## Methods

### Identification and copy number determination of *IC*

The *IC* family was first identified in *R. marina* by TBLASTN searching with the DD34E references (*Passport*, *Prince*, *Quetzal*, and *Sleeping beauty*), then its taxonomic distribution was investigated by a TBLASTN search against all the available organism genomes deposited at the NCBI database using the *IC R. marina* transposase (346 aa) as query. The *IC* transposon was considered to be present in one species when a unique DD36E motif of the transposon catalytic domain was detected. The obtained *IC* transposases were in turn used as queries to identify more *IC* elements. To determine the boundaries of these elements in each species, the best hits were extracted with a 2 kb flanking sequences, aligned using the ClustalW program within the BioEdit tool [46], and the transposon boundaries were then checked manually. In addition, copies (> 10) in each species were also aligned using the ClustalW program within the BioEdit tool [46] and their consensus sequences were reconstructed using the above multiple alignments in each genome using DAMBE after gaps were removed [47]. If one genome sequence contained a low copy number (< 10), the best hit was used as the representative sequence of *IC* in this species. Then, these consensus sequences were entered into BLASTN for each host genome to estimate copy numbers. All BLAST hits with more than 1000 bp in size and 90% identity were used to calculate copy numbers. The copy of the transposon possessing the complete TIR sequence and encoding the entire or longest transposase was used as a representative sequence for the transposon in the species for further structural organization and polygenetic analysis.

### Sequence analyses

TIRs were manually checked by using the BioEdit tool [46]. The potential ORF of *Incomer* used in the present study was predicted by Genscan (http://hollywood.mit.edu/GENSCAN.html). Protein secondary structures of *IC*-encoded transposases were predicted using PSIPRED [48]. Putative NLS motifs were predicted using PSORT II Prediction as provided in the PSORT Internet server (http://psort.nibb.ac.jp/). Multiple alignments of these elements were created by MUSCLE [49]. Shading and minor manual refinements of these aligned sequences were deduced using GeneDoc [50]. The pairwise divergence between elements and the average divergence from the consensus sequence were calculated using Kimura’s 2-parameter method in MEGA software v. 7.2.06 [51]. Sequence identities between *IC* family and eight other families were measured with the pairwise comparisons of full-length transposases by using the BioEdit tool [46]. And sequence identities between *IC* elements among species were measured by pairwise comparisons of full-length *IC* consensus sequences, and *ICs* in 10 species in cluster B were selected as the representatives for this analysis.

### Phylogenetic and HT analyses

The conserved DDE domain of the identified *IC* transposases and full-length transposases were aligned to the 28 known DNA transposases representing eight families from *Tc1/mariner* superfamily separately by MAFFT v 7.310 [26] (Additional file 4: Text S1 and Additional file 5: Text S2). The species that only had highly fragmented copies and incomplete DD36E motifs in their genome were not included in this analysis. Transposase sequences of DD34E/*Tc1*, DD34D/*Mariner*, DD37D/*maT*, DD39D, DD41D, DD35E, DD × D/*pogo* and DD37E/*TRT* were downloaded from GenBank. The best-suited aa substitution model for these data was the VT + G4 model according to BIC which were selected by ModelFinder embed in IQ-TREE program [27, 52]. Bootstrapped (1000 replicates) phylogenetic trees were inferred by using the maximum-likelihood method in IQ-TREE (v. 1.6.1) [27] .

Coding sequence of *RAG1*genes were used in the comparison with transposon distance, with the purpose of testing HT hypothesis. Their accession numbers were listed in (Additional file 6: Table S3). Species that cannot find the complete CDS region of the *RAG1* gene in NCBI database are not included in this calculation. Multiple alignments of *RAG1* and *IC* were created using MUSCLE [49]. Then, comparison distances of *RAG1* and *IC* were calculated using MEGA software v. 7.2.06 [51] (pairwise deletion, maximum composite likelihood) based on two aligned files (Additional file 7: Text S3 and Additional file 8: Text S4).

## Supplementary information

**Additional file 1: Table S1.** Taxonomic distribution of *Incomer*.

**Additional file 2: Figure S1–5. Figure S1:** Taxonomic distribution of *IC* elements in Actinopterygii. **FigureS2:** Alignment of domains of Incomer and DD34E/*Tc1* transposases. **FigureS3:** Full tree of *IC* elements with eight other members of the *Tc1/mariner* superfamily based on their full-length transposases **FigureS4:** Full tree of *IC* elements with eight other members of the *Tc1/mariner* superfamily based on their DDE/D motifs. **FigureS5:** Time tree of species harboring *IC* elements.

**Additional file 3: Table S2.** Distance of *IC* elements and *RAG1* genes and divergence time of each species using pairwise comparisons. (XLS 52 kb)

**Additional file 4: Text S1.** Sequence alignment of transposase DDE/D domains used to calculate the tree.

**Additional file 5: Text S2.** Sequence alignment of full-length transposase used to calculate the tree.

**Additional file 6: Table S3.** Access number of *RAG1* genes. Species only with the complete CDS region of the *RAG1* gene in the NCBI database are listed.

**Additional file 7: Text S3**. Alignment of CDS regions of *RAG1* genes.

**Additional file 8: Text S4**. Alignment of *IC* elements.

## Data Availability

All data generated or analysed during this study are included in this published article and its supplementary information files.

## Abbreviations

bp: base pairs
DBD: DNA-binding domain
HTH: Helix–turn–helix
Ma: Million years ago
MITEs: Miniature inverted-repeat transposable elements
NLS: Nuclear localization sequence
*RAG1*: Recombination-activating gene 1
TEs: Transposable elements
TIRs: Terminal inverted repeats
