# Supplementary material for: Incomer, a DD36E family of Tc1/mariner transposons newly discovered in animals
Source: Mob DNA. 2019 Nov 23;10:45. doi: 10.1186/s13100-019-0188-x (PMC6875036; doi:10.1186/s13100-019-0188-x)
Supplement: Supplementary file 2 — Additional file 2: Figure S1–5. Figure S1: Taxonomic distribution of IC elements in Actinopterygii. FigureS2: Alignment of domains of Incomer and DD34E/Tc1 transposases. FigureS3: Full tree of IC elements with eight other members of the Tc1/mariner superfamily based on their full-length transposases FigureS4: Full tree of IC elements with eight other members of the Tc1/mariner superfamily based on their DDE/D motifs. FigureS5: Time tree of species harboring IC elements. [file 13100_2019_188_MOESM2_ESM.docx]

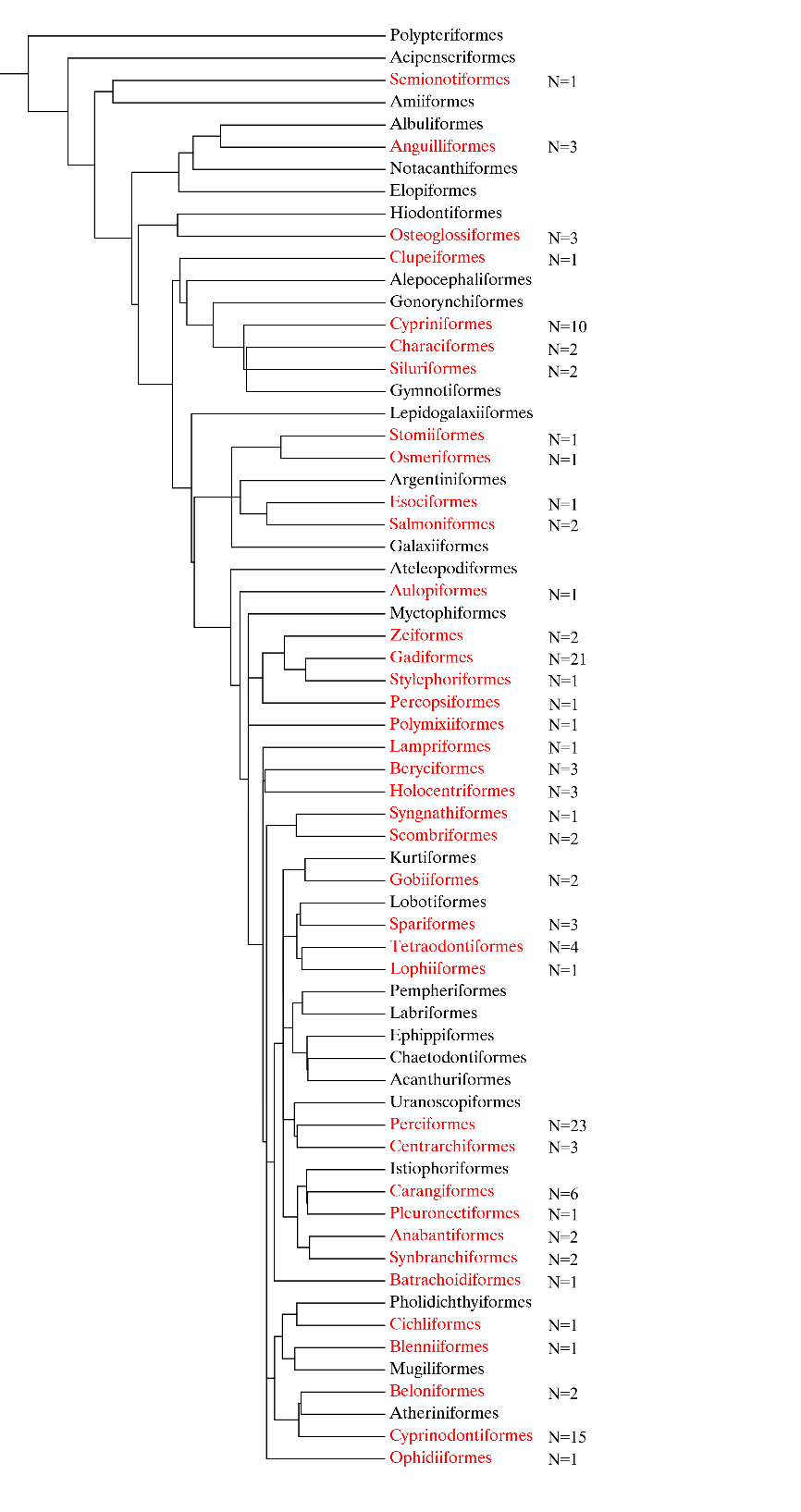


**Figure S1:** Taxonomic distribution of *IC* elements in Actinopterygii. The taxonomic tree represents the distribution of 132 species identified in the Actinopterygii (ray-finned fish) in their respective orders. Orders harboring *IC* elements are shown in red. The number of species harboring *IC* elements is shown in the right.


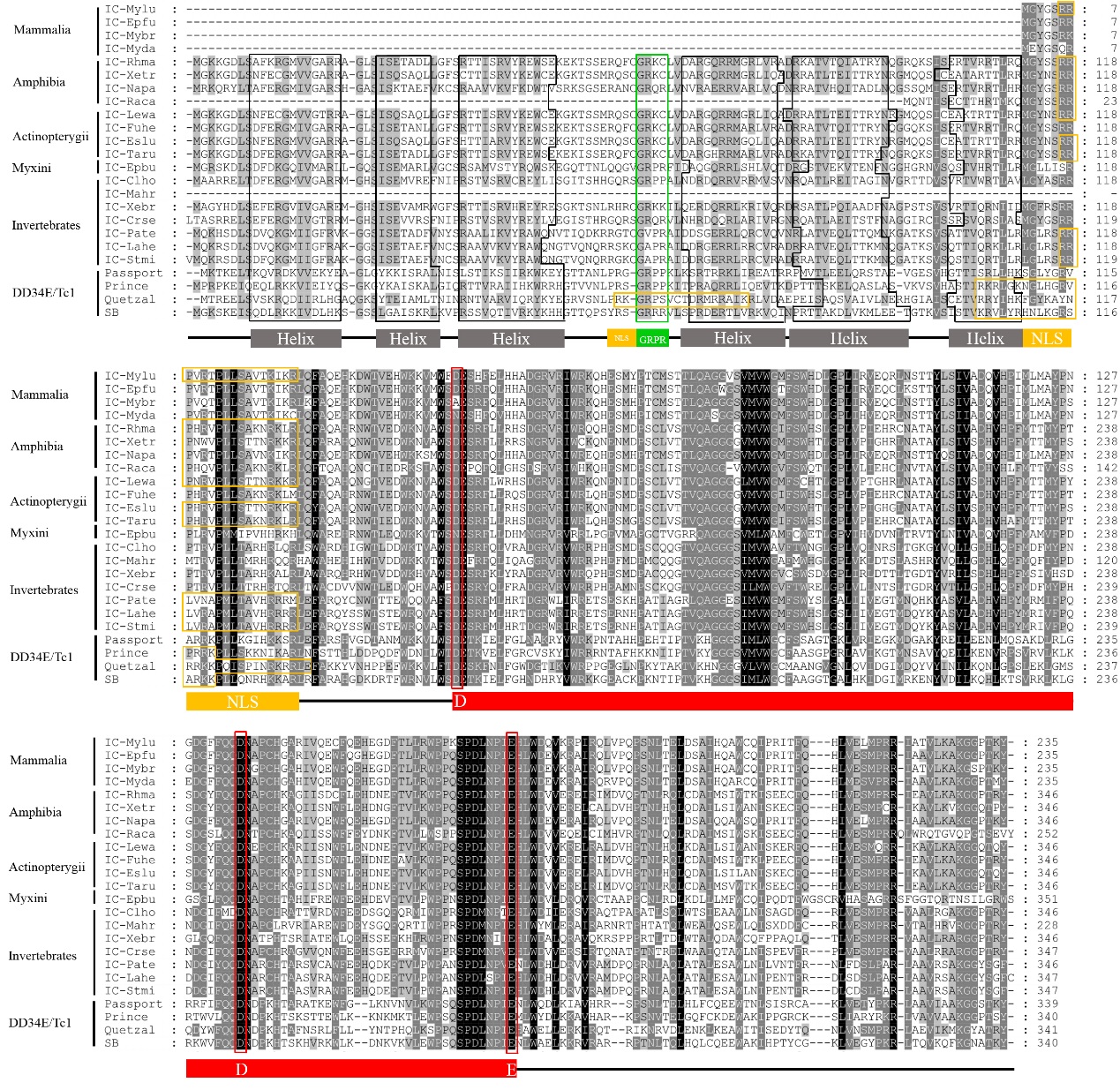


**Figure S2:** Alignment of domains of Incomer and DD34E/*Tc1* transposases. Only 20 representative species were used in this analysis, including the Mammalia (four species), Amphibia (four species), Actinopterygii (four species), Myxini (one species) and Invertebrates (seven species). Four respective transposases (*Passport*, *Prince*, *Quetzal* and *Sleeping beauty*) of DD34E/*Tc1* family were used as reference.





**Figure S3:** Full tree of *IC* elements identified in this study with eight other families of the *Tc1/mariner* superfamily based on the alignment of the full-length transposases.

**
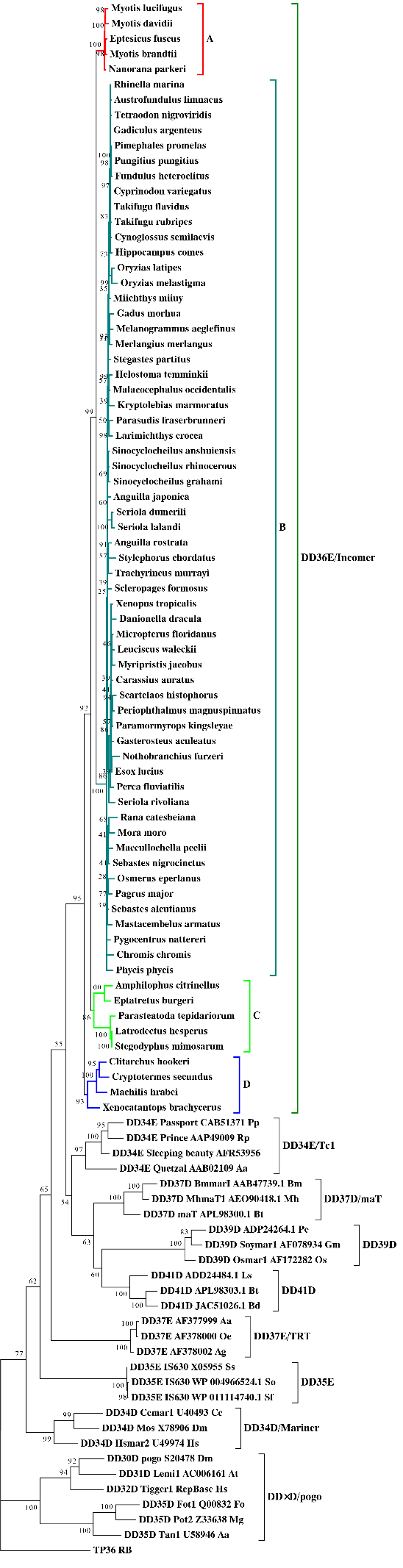
**

**Figure S4:** Full tree of *IC* elements identified in this study with eight other families of the *Tc1/mariner* superfamily based on their DDE/D motifs. Species with only highly fragmented copies and incomplete DD36E motifs in their genome were not included in this analysis.


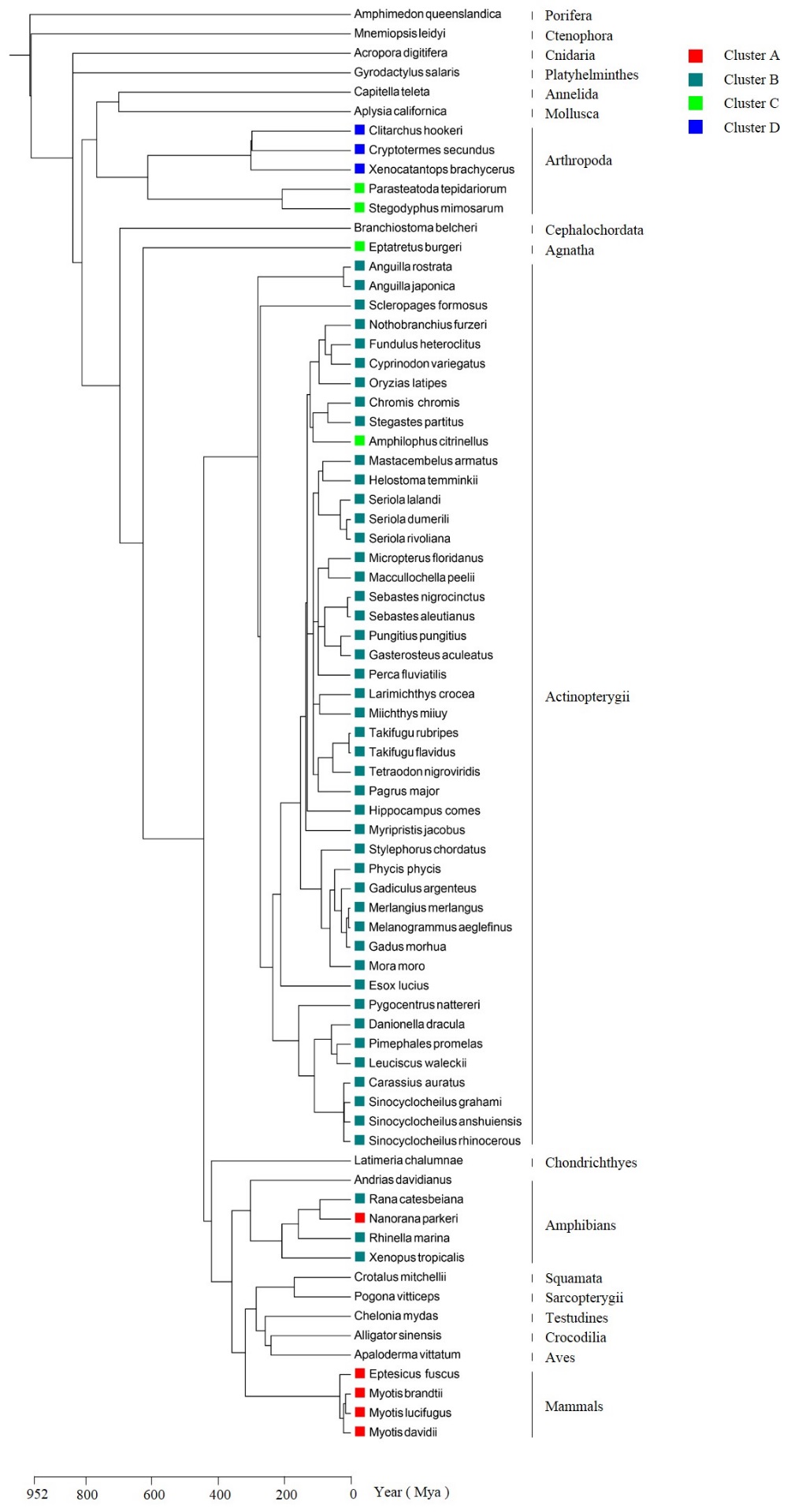


**Figure S5:** Time tree of species harboring *IC* elements identified in this study. The phylogenetic relationships and divergence times are taken from the TimeTree database (http://timetree.org/)
