# Supplementary material for: Incomer, a DD36E family of Tc1/mariner transposons newly discovered in animals
Source: Mob DNA. 2019 Nov 23;10:45. doi: 10.1186/s13100-019-0188-x (PMC6875036; doi:10.1186/s13100-019-0188-x)
Supplement: Supplementary file 6 — Additional file 6: Table S3. Access number of RAG1 genes. Species only with the complete CDS region of the RAG1 gene in the NCBI database are listed. [file 13100_2019_188_MOESM6_ESM.pdf]

**Species**	**RAG1**
Pygocentrus nattereri	XM_017700009.1
Sinocyclocheilus grahami	XM_016267534.1
Seriola lalandi dorsalis	XM_023406681.1
Seriola dumerili	XM_022754679.1
Esox lucius	XM_010883748.1
Carassius auratus	EF186007.3
Paramormyrops kingsleyae	XM_023842066.1
Nothobranchius furzeri	XM_015964816.1
Mastacembelus armatus	XM_026310413.1
Cyprinodon variegatus	XM_015389859.1
Takifugu rubripes	XM_003967412.2
Fundulus heteroclitus	XM_012853224.2
Cynoglossus semilaevis	XM_025056486.1
Hippocampus comes	XM_019893918.1
Austrofundulus limnaeus	XM_014005535.1
Oryzias melastigma	XM_024282789.1
Oryzias latipes	XM_023955935.1
Larimichthys crocea	XM_010738854.3
Stegastes partitus	XM_008275863.1
Kryptolebias marmoratus	XM_017423079.2
Myotis brandtii	XM_005872642.2
Eptesicus fuscus	XM_008146798.2
Myotis davidii	XM_006756100.2
Myotis lucifugus	XM_023763518.1
